# Aspirin for primary prevention of cardiovascular disease: a meta-analysis with a particular focus on subgroups

**DOI:** 10.1186/s12916-019-1428-0

**Published:** 2019-11-04

**Authors:** Georg Gelbenegger, Marek Postula, Ladislav Pecen, Sigrun Halvorsen, Maciej Lesiak, Christian Schoergenhofer, Bernd Jilma, Christian Hengstenberg, Jolanta M. Siller-Matula

**Affiliations:** 1 Department of Clinical Pharmacology, Medical University of Vienna, Vienna, Austria; 2 Department of Experimental and Clinical Pharmacology, Centre for Preclinical Research and Technology (CEPT), Medical University of Warsaw, Warsaw, Poland; 3 Institute of Computer Science of Academy of Sciences of the Czech Republic, Prague, Czech Republic; 4 Department of Cardiology, Oslo University Hospital, Oslo, Norway; 5 1st Department of Cardiology, Poznan University of Medical Sciences, Poznan, Poland; 6 Division of Cardiology, Department of Internal Medicine II, Medical University of Vienna, Währinger Gürtel 18-20, 1090 Vienna, Austria

**Keywords:** Primary prevention, Aspirin, Cardiovascular disease, Major adverse cardiovascular event, Myocardial infarction, Stroke, Major bleeding, Cancer, Meta-analysis

## Abstract

**Background:**

The role of aspirin in primary prevention of cardiovascular disease (CVD) remains unclear. We aimed to investigate the benefit-risk ratio of aspirin for primary prevention of CVD with a particular focus on subgroups.

**Methods:**

Randomized controlled trials comparing the effects of aspirin for primary prevention of CVD versus control and including at least 1000 patients were eligible for this meta-analysis. The primary efficacy outcome was all-cause mortality. Secondary outcomes included cardiovascular mortality, major adverse cardiovascular events (MACE), myocardial infarction, ischemic stroke, and net clinical benefit. The primary safety outcome was major bleeding. Subgroup analyses involving sex, concomitant statin treatment, diabetes, and smoking were performed.

**Results:**

Thirteen randomized controlled trials comprising 164,225 patients were included. The risk of all-cause and cardiovascular mortality was similar for aspirin and control groups (RR 0.98; 95% CI, 0.93–1.02; RR 0.99; 95% CI, 0.90–1.08; respectively). Aspirin reduced the relative risk (RRR) of major adverse cardiovascular events (MACE) by 9% (RR 0.91; 95% CI, 0.86–0.95), myocardial infarction by 14% (RR 0.86; 95% CI, 0.77–0.95), and ischemic stroke by 10% (RR 0.90; 95% CI, 0.82–0.99), but was associated with a 46% relative risk increase of major bleeding events (RR 1.46; 95% CI, 1.30–1.64) compared with controls. Aspirin use did not translate into a net clinical benefit adjusted for event-associated mortality risk (mean 0.034%; 95% CI, − 0.18 to 0.25%). There was an interaction for aspirin effect in three patient subgroups: (i) in patients under statin treatment, aspirin was associated with a 12% RRR of MACE (RR 0.88; 95% CI, 0.80–0.96), and this effect was lacking in the no-statin group; (ii) in non-smokers, aspirin was associated with a 10% RRR of MACE (RR 0.90; 95% CI, 0.82–0.99), and this effect was not present in smokers; and (iii) in males, aspirin use resulted in a 11% RRR of MACE (RR 0.89; 95% CI, 0.83–0.95), with a non-significant effect in females.

**Conclusions:**

Aspirin use does not reduce all-cause or cardiovascular mortality and results in an insufficient benefit-risk ratio for CVD primary prevention. Non-smokers, patients treated with statins, and males had the greatest risk reduction of MACE across subgroups.

**Systematic review registration:**

PROSPERO CRD42019118474.

## Background

Acetylsalicylic acid (commonly referred to as “aspirin”) is an antithrombotic agent that inhibits platelets by irreversibly acetylating the serine residue of cyclooxygenase-1 (COX-1) in platelets with subsequently reduced levels of prothrombotic thromboxane A_2_ (TxA_2_) [1–3]. In patients with known cardiovascular disease (CVD), the potential for aspirin to reduce further cardiovascular (CV) events significantly outweighs the risks of major bleeding and thus aspirin has since become a mainstay in secondary prevention of CVD [4–8]. However, in primary prevention, its role is still under debate [9]. This is due to an as yet unclear balance between the benefits and risks of aspirin treatment in patients without a diagnosed atherosclerotic disease.

Previously published meta-analyses have indicated that aspirin significantly reduced myocardial infarction (MI) and major adverse cardiovascular events (MACE) without an impact on stroke and CV- or all-cause death [10–14]. Furthermore, an increased risk of major bleeding events under aspirin strongly outweighed the benefits of aspirin treatment in primary prevention [10, 12–14]. As a result, the current guidelines on CVD prevention from the European Society of Cardiology (ESC) do not recommend antiplatelet therapy in patients free of overt CVD [8]. On the contrary, the recently published 2019 ACC/AHA guideline on the primary prevention of cardiovascular disease states that aspirin might be considered in selected adults aged 40 to 70 who are at higher CV risk but at no increased bleeding risk [15]. The U.S. Preventive Services Task Force recommends initiation of aspirin treatment depending on age and 10-year CVD risk [16].

Recently, three major trials (ARRIVE, ASCEND, and ASPREE) evaluating the use of aspirin in primary prevention of CVD were published [17–19]. The ARRIVE trial enrolled patients with moderate to high cardiovascular risk, the ASCEND trial patients with diabetes mellitus (DM) only, and the ASPREE trial elderly patients. Only the ASCEND trial [18] showed a significant reduction in the rate of major adverse CV events, but the effect was, once again, accompanied by a significant increase in major bleeding. Using the three recently published trials, we aimed to perform a meta-analysis with a particular focus on subgroups in order to potentially characterize patient populations with a more favorable benefit-risk ratio.

## Methods

### Protocol and registration, data extraction, and quality assessment

Our review was registered with PROSPERO under the registration number CRD42019118474. Two reviewers applied the selection criteria (GG and JMSM) independently and in duplicate. This study was conducted in accordance with Preferred Reporting Items for Systematic Reviews and Meta-Analyses (PRISMA) guidelines, as described previously [20–24].

### Data sources and searches

We searched PubMed and Web of Science using predefined search terms (primary prevention AND aspirin AND clinical trial OR meta-analysis) until November 2018. Six additional trials [25–30] that were included in a previous meta-analysis [5] were also identified and included in our analysis. The titles and abstracts of suspected relevant citations were screened for eligibility, and the full text was acquired for further evaluation if the citation was deemed pertinent. References of retrieved meta-analyses and reviews were also checked for additional trials.

### Study selection and outcomes

Included studies had to be randomized controlled trials (RCT) and include at least 1000 patients. Studies had to be controlled (placebo or control group), but could be open-label or blinded. The target patient population comprised patients without any history of CVD. Patients with a low ankle-brachial index (ABI) who had no symptoms and no diagnosis of peripheral arterial disease were considered as a primary prevention cohort. Exclusion criteria were non-RCTs, duplicate reports, ongoing studies, and studies that included patients with history of CVD.

The primary efficacy outcome was all-cause mortality. Secondary efficacy outcomes included cardiovascular mortality, the composite of major adverse cardiovascular events (MACE), MI, and ischemic stroke (IS). MACE was defined as a composite of nonfatal stroke, nonfatal MI, and CV mortality. In order to accurately assess the rate of MACE, we performed two analyses, one comparing the calculated rate of MACE as per our definition and one comparing the rate of the study defined primary outcome as a part of a sensitivity analysis. Stroke was defined as “ischemic stroke” but not all included studies reported on the incidence of IS alone. If not sufficiently specified, the number of reported strokes was used. We also reported on the incidence of hemorrhagic stroke. Bearing in mind the uncertain effect of aspirin on cancer outcomes, cancer risk was prespecified as an exploratory outcome. For further analysis of data, we performed four subgroup analyses involving diabetes, sex, concomitant statin treatment, and smoking.

Major bleeding was the primary safety endpoint. Definition of major bleeding varied between studies. If not defined as “major bleeding,” we used the following definitions: “bleeding requiring transfusion,” “bleeding rendering patients intensive care dependent,” “bleeding causing death,” or “intracranial bleeding.” The extracranial major bleeding analysis comprised the total of all major bleedings and some GI bleeding events that were classified as relevant in respect to the analysis. Intracranial hemorrhages and GI bleedings were also assessed as single endpoints.

### Data synthesis and analysis

Variables are reported as numbers or percentages as appropriate. Risk ratios (RR) were calculated from individual studies and pooled according to the inverse variance model with 95% confidence intervals (95% CI) and reported as relative risk reduction or increase respectively (RRR/RRI) within a mean time frame of 6.4 years (which is the mean follow-up period of included studies). The statistical inconsistency test (*I*^2^) was used to assess heterogeneity vs. homogeneity between studies. If the *I*^2^ value was low (*I*^2^ < 50%), a fixed-effect model was additionally calculated, as reported previously [20, 22, 24]. The following sensitivity analyses were performed: (i) comparison of the results of the fixed vs. random-effect model, (ii) the influence of each study was assessed by testing whether deleting each in turn would have significantly changed the pooled results of the meta-analysis, (iii) sensitivity analysis of the date of publication before and after 2010, (iv) sensitivity analysis assessing the length of follow-up (< 5 vs. > 5 years), and (v) and analysis focusing on the study defined primary outcome parameter.

Absolute risk reduction or increase (ARR, ARI) and number needed to treat or harm (NNT, NNH) were calculated per 1 year of treatment. This was performed as follows: event incidence rates were divided by their respective mean follow-up periods and multiplied by 100 to obtain the incidence rate per 100 patient years. Out of these, the ARR or ARI were calculated by subtraction, and subsequently, the NNT or NNH were calculated according to the following formula: NNT or NNH = 1/(ARR or ARI). Events prevented/caused per 10,000 patients per year were calculated by dividing 10,000 by the NNT or NNH. This transformation of data allows for a better understanding of risks for doctors and patients.

The mortality-adjusted net clinical benefit was calculated as follows: [IR_ischemi stroke_aspirin_ + w1IR_myocardial infarction_aspirin_ + w2IR_hemorrhagic stroke_aspirin_ + w3IR_major extracranial bleeding_aspirin_] − [IR_ischemic stroke_control_ + w1IR_myocardial infarction_control_ + w2IR_hemorrhagic stroke_control_ + w3IR_major extracranial bleeding_control_], where w1, w2, and w3 are the death-related weights associated with each type of event. Weights were calculated as the impact of each event on mortality, as derived from recent analyses [31, 32], and related to IS (weight = 1). Weights were thus w1 = 0.89 for MI, w2 = 3.23 for hemorrhagic stroke, and w3 = 0.63 for major extracranial bleeding [31, 32]. In the mortality-adjusted net clinical benefit analysis, a lower estimate indicates a greater benefit of aspirin.

A two-tailed *p* value of < 0.05 was considered significant. Review Manager (Version 5.3. Copenhagen: The Nordic Cochrane Centre, The Cochrane Collaboration, 2014) was used for statistical calculations.

## Results

### Description of studies

Our search retrieved 608 references. Five hundred ninety items were excluded based on title and abstracts that were not RCTs, investigated aspirin in secondary prevention of CVD, or were identified as non-pertinent studies (Additional file 1: Figure S1). Additionally, retrieved reviews and meta-analyses were thoroughly examined to identify further trials. One study was excluded as it contained a significant number of patients with definite or suspected CVD [33]. Thirteen trials [17–19, 25–30, 34–37] were eligible for analysis and comprised a total of 164,225 patients, 82,900 allocated to aspirin and 81,325 allocated to the control group. One included study [36] was a 10-year follow-up of a previously published trial [38]. The mean age of patients included in our meta-analysis was 62 years. The mean follow-up period was 6.4 years (ranged from 3.6 to 10.3 years). Three trials exclusively included patients with known diabetes [18, 36, 37]. Three trials included men only [25, 28, 30], and one trial included women only [29]. The dosage of aspirin ranged from 75 to 500 mg once daily. Two trials evaluated the effect of aspirin (325 mg and 100 mg) given on alternate days [29, 30]. Only two studies reported the use of proton-pump inhibitors (PPIs) [18, 19]. Included studies are characterized in Tables 1 and 2.

**Table 1:** Characteristics of included studies

Study	Study design	Study population	Inclusion criteria	Exclusion criteria	Median follow-up	Primary endpoint	Aspirin treatment regimen	*N*	Placebo-controlled
Peto et al. [28] (British Doctors’ Study)	Open-label RCT	Male British doctors	Male doctors residing in the UK and listed in the 1977 Medical Directory who previously replied to a questionnaire about their smoking habits	-Ongoing aspirin use -History of peptic ulcer, strike or myocardial infarction	6 years	Myocardial infarction, stroke, transient ischemic attacks	500 mg once daily	5139	No
Steering Committee of the Physicians’ Health Study Research Group [30] US Physicians’ Health Study 1989 (PHS)	Double-blind RCT	Healthy male doctors	-Male physician -40 to 84 years of age	-History of myocardial infarction, stroke, transient ischemic attack, cancer (except nonmelanoma skin cancer) -Current liver or kidney disease -Peptic ulcer -Gout -Contraindications to aspirin consumption -Current use of aspirin, other platelet-active drugs, nonsteroidal anti-inflammatory drugs, or current use of vitamin A supplement	5 years	Myocardial infarction, stroke, cardiovascular mortality	325 mg on alternate days	22,071	Yes
The Medical Research Council’s General Practice Research Framework [25] Thrombosis Prevention Trial 1998 (TPT)	Double-blind RCT	Men at high risk of IHD	-Males between age 46 and 69 -Top 20% of risk score distribution -Top 25% in regions with particularly high ischemic heart disease (IHD) mortality rates	-Peptic ulceration -History of possible or definite myocardial infarction or stroke -Medication not compatible with trial treatment	6.8 years	All IHD (sum of coronary death and fatal and nonfatal myocardial infarction)	75 mg once daily	5085	Yes
Hansson et al. [27] (Hypertension Optimal Treatment Trial, HOT)	Double-blind RCT	Men and women with hypertension	-Age 50 to 80 years -Hypertension -Diastolic blood pressure between 100 and 115 mmHg	-Suspicion of incorrect inclusion or data handling	3.8 years	MACE was defined as all (fatal and nonfatal) myocardial infarctions and strokes and all other cardiovascular deaths	75 mg once daily	18,790	Yes
Collaborative Group of the Primary Prevention Project [26] Primary Prevention Project (PPP) 2001	Open-label RCT	Men and women with one or more risk factors for coronary heart disease (CHD)	-Age > 65 years -Hypertension (SBP ≥ 160, DBP ≥ 95) -Hypercholesterolemia -Diabetes mellitus -Obesity -Family history of myocardial infarction before 55 years of age in at least one parent or sibling	-Treatment with antiplatelet drugs, chronic -Chronic use of anti-inflammatory drugs or anticoagulants -Contraindications to aspirin -Disease with predictable poor short-term prognosis -Predictable psychological or logistical difficulties affecting compliance with the trial requirements	3.6 years	Cumulative rate of cardiovascular death, nonfatal myocardial infarction, and nonfatal stroke	100 mg once daily	4495	No (vitamin E)
Ridker et al. [29] (Women’s Health Study, WHS)	Double-blind RCT	Females aged 45 and over	-Women aged ≥ 45	-No history of coronary heart disease, cerebrovascular disease, cancer (except nonmelanoma skin cancer), other major chronic illnesses -History of side-effects to any of the study medications -Intake of aspirin or nonsteroidal anti-inflammatory drugs more than once a week -Use of anticoagulants or corticosteroids -Intake of supplements of vitamins A and E and beta-carotene more than once a week	10.1 years	Nonfatal myocardial infarction, nonfatal stroke, or death from any cardiovascular causes	100 mg on alternate days	39,876	Yes
Belch et al. [37] (POPADAD trial)	Double-blind RCT	Diabetic patients aged 40 and over with a reduced ABI	-Men and women aged ≥ 40 -Diagnosis of type I or II diabetes mellitus -Asymptomatic peripheral artery disease (ABI ≤ 0.99)	-Evidence of symptomatic cardiovascular disease -Use of aspirin or antioxidant therapy -Peptic ulceration, severe dyspepsia -Bleeding disorder -Intolerance to aspirin -Suspected serious physical illness with reduced life expectancy -Congenital heart disease -Psychiatric illness	6.7 years	Two hierarchical composite primary endpoints: (1) death from coronary heart disease or stroke, nonfatal myocardial infarction or stroke, or above ankle amputation for critical limb ischemia and (2) death from coronary heart disease or stroke	100 mg once daily	1276	Yes
Ogawa et al. [38] (Saito et al. [36], 10-year follow-up) (JPAD trial)	Open-label RCT	Patients with diabetes	-Age between 30 and 85 years -Type 2 diabetes mellitus -Ability to give informed consent	-Ischemic ECG abnormalities -History of CHD -History of coronary angiography -History of cerebrovascular disease -History of arteriosclerotic disease requiring treatment -Atrial fibrillation -Pregnancy -Use of antiplatelet/antithrombotic therapy -History of severe gastric or duodenal ulcer -Severe renal or liver dysfunction -Allergy to aspirin	4.4 years/10.3 years	Any atherosclerotic event, which was a composite of sudden death, death from coronary, cerebrovascular or aortic causes, nonfatal acute myocardial infarction, unstable angina, newly developed exertional angina, nonfatal ischemic and hemorrhagic stroke, transient ischemic attack, nonfatal aortic or disease	81–100 mg once daily	2539	No
Fowkes et al. [34] (Aspirin for Asymptomatic Atherosclerosis trial, AAA)	Double-blind RCT	Men and women with a low ABI	-Men and women aged 50 to 75 years -No history of vascular disease -ABI ≤ 0.95	-History of myocardial infarction, stroke, angina, or peripheral artery disease -Use of antiplatelet/antithrombotic therapy -Severe renal or liver dysfunction -Severe indigestion -Receiving chemotherapy -High/low hematocrit -Contraindication to aspirin	8.2 years	Composite of initial fatal or nonfatal coronary event or stroke or revascularization	100 mg once daily	3350	Yes
Ikeda et al. [35] (Japanese Primary Prevention Trial, JPPP)	Open-label RCT	Men and women with one or more risk factors for CHD	-Men and women aged 60 to 85 years -No atherosclerotic disease -Hypertension (SBP ≥ 140, DBP ≥ 90) -Dyslipidemia -Diabetes mellitus	-History of coronary artery disease -History of cerebrovascular disease -Atherosclerotic disease requiring surgery -Atrial fibrillation -Peptic ulcer or conditions associated with bleeding -Aspirin-sensitive asthma and history of aspirin allergy -Receiving antiplatelet agents, anticoagulants, or nonsteroidal anti-inflammatory drugs	5 years	Composite of death from cardiovascular causes (myocardial infarction, stroke, and other cardiovascular causes), nonfatal stroke, and nonfatal myocardial infarction	100 mg once daily	14,464	No
Gaziano et al. [17] (ARRIVE trial)	Double-blind RCT	Men and women with multiple risk factors for CHD	-Males aged 55 years and older with between 2 and 4 risk factors -Females aged 60 years and older with between 3 and 4 risk factors -Risk factors were high cholesterol or LDL -Current smoking -Low HDL cholesterol -High blood pressure -Receiving medication for high blood pressure -Positive family history of cardiovascular heart disease	-Diabetes mellitus -History of a vascular event, such as stroke, myocardial infarction, coronary artery angioplasty, or stenting -Coronary artery bypass graft -Relevant arrhythmias -Congestive heart failure -Vascular intervention -Ongoing antiplatelet therapy -High risk of gastrointestinal and other bleeding, including those with a history of gastric or duodenal ulcers or gastrointestinal bleeding -Requiring concomitant use of anticoagulants or frequent use of nonsteroidal anti-inflammatory drugs	5 years	Composite outcome of confirmed myocardial infarction, stroke, cardiovascular death, unstable angina, or transient ischemic attack	100 mg once daily	12,546	Yes
The ASCEND Study Collaborative Group [18] (ASCEND trial) 2018	Single-blind RCT	Diabetic men and women aged ≥ 40	-Men and women with at least 40 years of age -Diagnosis of diabetes mellitus -No known cardiovascular disease	-Clear indication for aspirin -Contraindication to aspirin -Presence of other clinically significant conditions that might limit adherence to the trial regimen for at least 5 years	7.4 years	First serious vascular event, which was defined as a composite of nonfatal myocardial infarction, nonfatal stroke (excluding confirmed intracranial hemorrhage) or transient ischemic attack, or death from any vascular cause (excluding confirmed intracranial hemorrhage)	100 mg once daily	15,480	Yes
McNeil et al. [19] (ASPREE trial)	Double-blind RCT	Elderly men and women	-Men and women aged ≥ 70 (≥ 65 in Hispanics and Latinos) -Free from overt coronary heart disease, overt cerebrovascular disease, atrial fibrillation, a clinical diagnosis of dementia, clinically significant physical disability, a high risk of bleeding, anemia, and a known contraindication to or inability to take aspirin	-Current regular use of an anticoagulant or antiplatelet medication other than aspirin -Systolic blood pressure of 180 mmHg or more or a diastolic blood pressure of 105 mmHg or more -A medical indication for or contraindication to regular aspirin therapy -Presence of a condition that, in the opinion of the primary care physician, was likely to result in death within 5 years	4.7 years	Prespecified secondary endpoint was cardiovascular disease, being a composite of fatal coronary heart disease, nonfatal myocardial infarction, fatal or nonfatal stroke, or hospitalization for heart failure	100 mg once daily	19,114	Yes

**Table 2:** Overview of study demographics

Study	Patients included	Mean age [years]	Mean follow-up [years]	% statin use	% PPI use	% diabetes	% male
British Doctors’ Study	5139	61	6	Unknown	Unknown	2	100
US Physicians’ Health Study	22,071	53	5	Unknown	Unknown	2	100
Thrombosis Prevention Trial	5085	57	6.8	Unknown	Unknown	2	100
Hypertension Optimal Treatment Trial	18,790	61	3.8	Unknown	Unknown	8	53
Primary Prevention Project	4495	64	3.6	Unknown	Unknown	17	43
Women’s Health Study	39,876	54	10.1	Unknown	Unknown	3	0
POPADAD trial	1276	60	6.7	Unknown	Unknown	100	44
JPAD trial	2539	65	10.3	26	8.6**	100	55
AAA trial	3350	62	8.2	Unknown	Unknown	3	28
JPPP trial	14,464	71	5	Unknown	Unknown	34	42
ARRIVE trial	12,546	64	5	Unknown	Unknown	0	70
ASCEND trial	15,480	63	7.4	75	Approx. 25	94.1	63
ASPREE trial	19,114	74*	4.7	34	24.7	11	44
all studies included	164,225	62	6.4 (IQR 4.85–7.8)				

*Median age

**Labeled as antiulcer medication

### Efficacy and safety outcomes

**Fig. 1:**
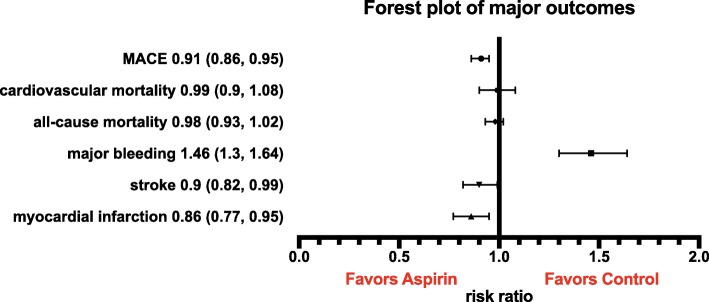
Risk ratios (RR) of the major outcomes

All trials reported on all-cause mortality [17–19, 25–30, 34–37] and included 164,225 patients. The incidence of all-cause mortality was similar between the aspirin and control groups (4.52% vs. 4.54%, respectively; RR 0.98; 95% CI, 0.93–1.02; *p* = 0.26; *I*^2^ = 0%; Fig. 1, Table 3, Additional file 1: Figure S2A). Use of aspirin was not associated with a reduction in CV mortality (RR 0.99; 95% CI, 0.90–1.08; *p* = 0.75; *I*^2^ = 0%; Fig. 1, Table 3; Additional file 1: Figure S3A) compared with no aspirin. Aspirin was associated with a RRR of MACE by 9% (RR 0.91; 95% CI, 0.86–0.95; *p* < 0.0001; *I*^2^ = 0%; ARR 0.052%; NNT 1908; Fig. 1, Additional file 1: Figure S2B), of MI by 14% (RR 0.86; 95% CI, 0.77–0.95; *p* = 0.005; *I*^2^ = 50%; ARR 0.041%; NNT 2452; Fig. 1, Table 3, Additional file 1: Figure S3B), and of IS by 10% (RR 0.90; 95% CI, 0.82–0.99; *p* = 0.03; *I*^2^ = 17%; ARR 0.022%; NNT 4448; Fig. 1, Table 3, Additional file 1: Figure S3C) compared with no aspirin.

**Table 3:** Risk estimates: absolute risk reduction (ARR) or increase (ARI) and number needed to treat (NNT) or to harm (NNH) for the primary and secondary endpoints over a treatment period of 1 year, which were statistically different between groups

Event	Events per 100 patient years in the aspirin group	Events per 100 patient years in the control group	ARR %	ARI %	NNT	NNH	Events prevented per 10,000 treated patients	Events caused per 10,000 treated patients	*p* value
Myocardial infarction	0.260	0.301	0.041		2452		4		0.005
Ischemic stroke	0.218	0.240	0.022		4448		2		0.03
MACE	0.613	0.665	0.052		1908		5		< 0.0001
Major bleeding	0.257	0.180		0.077		1295		8	< 0.0000
Extracranial major bleeding	0.286	0.218		0.068		1462		7	< 0.0000
GI bleeding	0.399	0.320		0.079		1263		8	< 0.0001

Twelve studies, including a total of 159,086 patients, reported on the rate of major bleeding complications [17–19, 25–27, 29, 30, 34–37]. Aspirin use was associated with a 46% RRI of major bleeding complications (RR 1.46; 95% CI, 1.30–1.64; *p* < 0.00001; *I*^2^ = 31%; ARI 0.077%; NNH 1295; Fig. 1, Table 3, Additional file 1: Figure S2C) compared with no aspirin. Extracranial major bleedings and GI bleedings were the major driver of the composite of bleeding events, with intracranial bleedings and hemorrhagic stroke having no statistical impact (Additional file 1: Figure S4). Aspirin did not decrease the cancer incidence (Additional file 1: Figure S5).

### The net clinical benefit

**Fig. 2:**
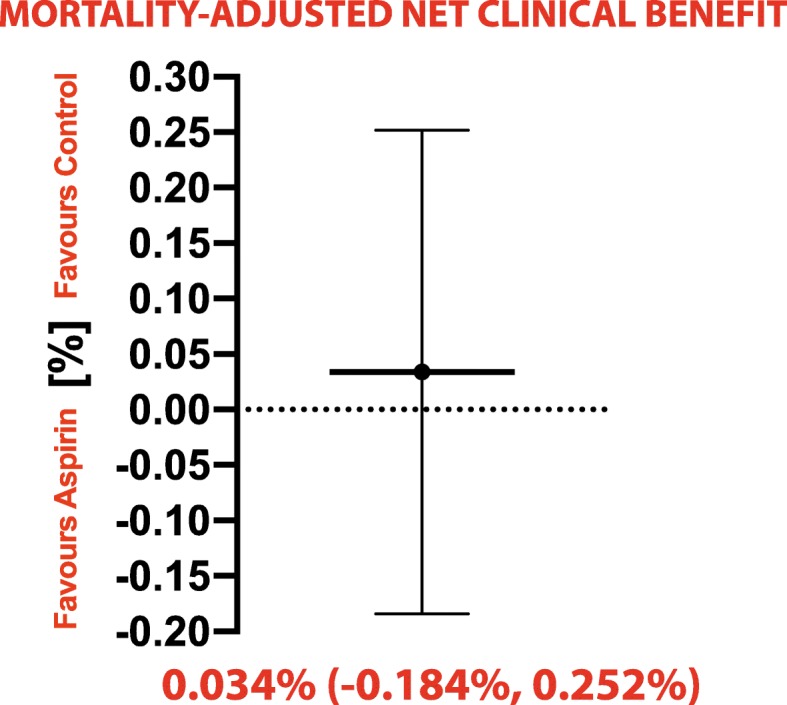
Analysis of the mortality-adjusted net clinical benefit

All trials [17–19, 25–30, 34–37] provided data for the estimation of the *adjusted* net clinical benefit. Aspirin was not associated with a net clinical benefit after adjustment for event-associated mortality risk (mean 0.034%; 95% CI, − 0.184 to 0.252%; Fig. 2).

The *crude* net clinical benefit comprising MACE and major bleeding events was calculated with data from 12 studies [17–19, 25–27, 29, 30, 34–37], showing that aspirin did not lead to a net clinical benefit (RR 1.01; 95% CI, 0.97–1.05; *p* = 0.54; *I*^2^ = 0%; Additional file 1: Figure S6).

### Analysis of subgroups

#### Statin treatment

##### MACE:

**Fig. 3:**
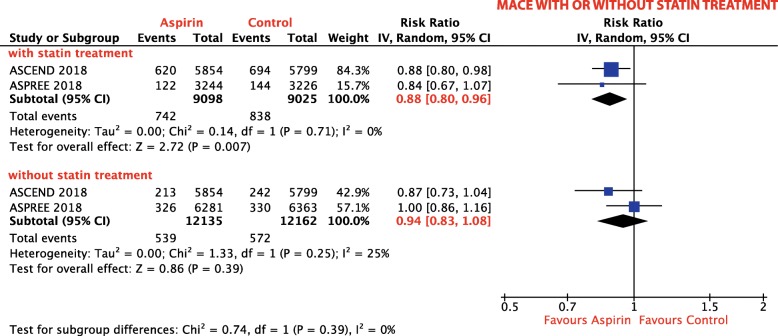
Subgroup analysis: Forest plot depicting the relative risk (RR) of MACE in patients with and without statin treatment

Two trials, comprising a total of 34,594 patients, reported on the MACE risk in patients with and without statin treatment [18, 19]. Aspirin-treated patients who were also treated with statins had a 12% RRR of MACE when compared with control plus statin (RR 0.88; 95% CI, 0.80–0.96; *p* = 0.007; *I*^2^ = 0%; Fig. 3). In contrast, aspirin without statin co-treatment did not reduce MACE risk when compared with control without statin (RR 0.94; 95% CI, 0.83–1.08; *p* = 0.39; *I*^2^ = 25%; Fig. 3).

#### Smoking

##### MACE:

**Fig. 4:**
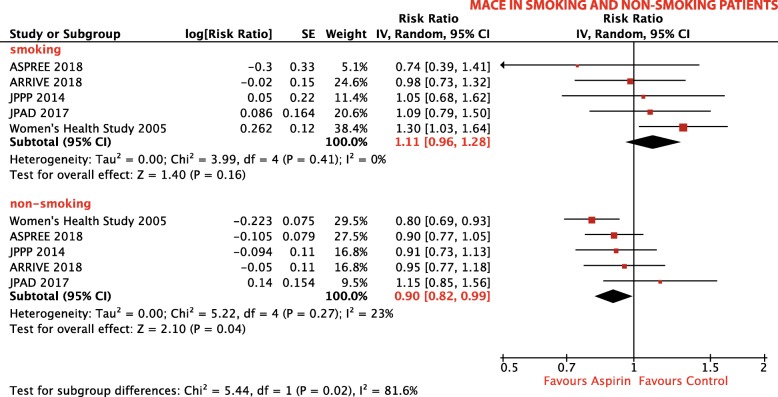
Subgroup analysis: Forest plot depicting the relative risk (RR) of MACE in smokers and non-smokers

Five trials, comprising a total of 88,539 patients, reported on incidence of MACE in smokers and non-smokers [17, 19, 29, 35, 36]. In non-smokers, aspirin use was associated with a 10% RRR of MACE (RR 0.90; 95% CI, 0.82–0.99; *p* = 0.04; *I*^2^ = 23%; Fig. 4) compared with no aspirin. In smokers, aspirin did not affect the risk of MACE (RR 1.11; 95% CI, 0.96–1.28; *p* = 0.16; *I*^2^ = 0%; Fig. 4).

#### Sex

##### MACE:

**Fig. 5:**
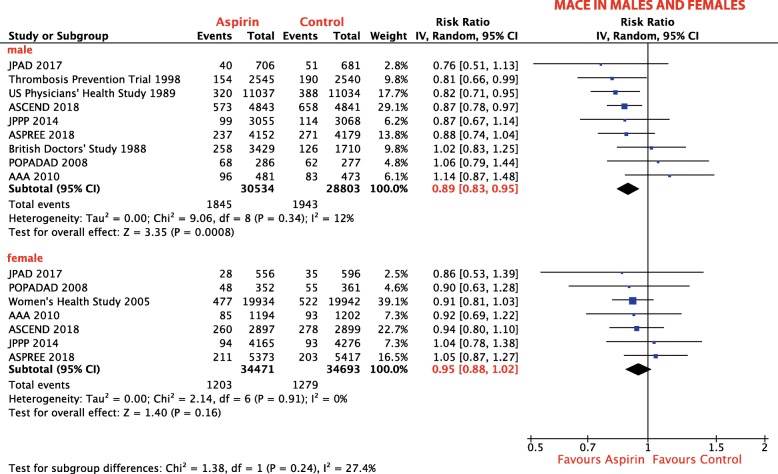
Subgroup analysis: Forest plot depicting the relative risk (RR) of MACE in males and females

Nine trials, including 59,337 patients, reported the incidence of MACE in men [18, 19, 25, 28, 30, 34–37], seven trials in women (69,164 patients) [18, 19, 29, 34–37]. There was a sex interaction for aspirin effect: the direction of the effect of aspirin on MACE tended to be similar in men and women, but the effect size differed, and did not reach statistical significance in females. Aspirin in men was associated with a RRR of MACE of 11% (RR 0.89; 95% CI, 0.83–0.95; *p* = 0.0008; *I*^2^ = 12%; Fig. 5) compared with controls. In women, aspirin did not significantly reduce the risk of MACE (RR 0.95; 95% CI, 0.88–1.02; *p* = 0.16; *I*^2^ = 0%; Fig. 5) compared with controls.

##### Myocardial infarction:

Three trials, including 32,295 patients, reported on MI in men [25, 28, 30], only one in women (39,876 patients) [29]. In men and in women, aspirin did not significantly reduce the risk of MI (RR 0.76; 95% CI, 0.57–1.01; *p* = 0.06; *I*^2^ = 76%; RR 1.03; 95% CI, 0.84–1.25; *p* = 0.26; respectively). This is in contrast to the overall population and underlines that the sex sub-analysis for MI is underpowered.

##### Ischemic stroke:

Three trials, including 32,295 patients, reported on IS in men [25, 28, 30], but only one trial reported these data for women (39,876 patients) [29]. Aspirin did not reduce the RR of IS in men (RR 1.02; 95% CI, 0.72–1.44; *p* = 0.93; *I*^2^ = 55%). In women, however, aspirin reduced IS by 23% (RR 0.77; 95% CI, 0.63–0.94; *p* = 0.010) compared to control as reported in one study.

#### Diabetes

##### MACE:

**Fig. 6:**
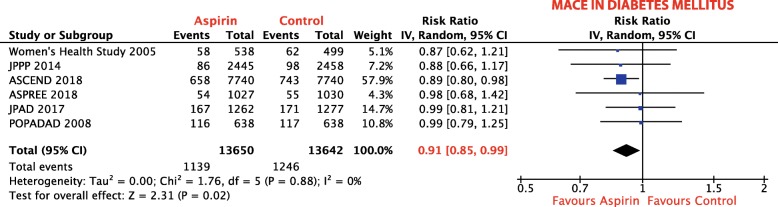
Subgroup analysis: Forest plot depicting the relative risk (RR) of MACE in patients with diabetes

Six studies, including 27,292 patients, reported on the rate of primary endpoint in patients with diabetes, showing a RRR of MACE by 9% (RR 0.91; 95% CI, 0.85–0.99; *p* = 0.02; *I*^2^ = 0%, Fig. 6), which is consistent with the analysis of the overall population. No data for a non-diabetic subgroup were available.

##### Myocardial infarction:

Five studies, comprising 20,865 patients, provided data for MI in diabetic patients, showing no difference between aspirin vs. control (RR 0.94; 95% CI, 0.72–1.23; *p* = 0.65; *I*^2^ = 53%) [18, 29, 30, 36, 37].

##### Ischemic stroke:

Four studies, including 20,332 patients, reported on IS in diabetic patients, showing a RRR of IS by aspirin of 24% (RR 0.76; 95% CI, 0.59–0.98; *p* = 0.03; *I*^2^ = 43%) [18, 29, 36, 37].

##### Sensitivity analyses:

Sensitivity analysis assessing the date of publication showed that the direction of the effect on MACE remained unchanged. However, the magnitude of the effect tended to be greater in studies published before 2010 compared to studies published after this date (RRR 11% vs. 7%, respectively).

Due to low heterogeneity (*I*^2^ = 0%), a fixed-effect model was calculated in addition to the random-effect model for each outcome (Additional file 1: Table S1), which confirmed the robustness of our findings.

By sequentially excluding one single study from the pooled analysis, the direction and the magnitude of the effect on MACE remained unchanged.

Sensitivity analysis assessing the length of follow-up/length of study drug use showed that the direction of effect on MACE remained unchanged. However, the magnitude of the effect tended to be greater in studies with a shorter-term use of aspirin (≤ 5 years, RRR 13%) vs. longer-term use (> 5 years, RRR 8%).

We additionally analyzed the primary endpoint of each study according to the study definition (which in some studies slightly differed from the MACE definition used in our meta-analysis). In the aspirin group, 4.3% of patients (3601/82,900) reached the primary endpoint compared to 4.7% in the control group (3827/81,325). Treatment with aspirin, therefore, significantly reduced the RR of the primary endpoint by 9% (RR 0.91; 95% CI, 0.87–0.95; *p* < 0.0001; *I*^2^ = 0%), confirming the result of the MACE analysis.

## Discussion

Our meta-analysis in over 160,000 patients without a history of CVD showed that aspirin did not reduce all-cause or CV mortality but reduced the risk of MACE, MI, and IS at the cost of an increased risk of major bleeding events. Hence, aspirin treatment was associated with a lower NNH than the NNT for the safety and efficacy outcomes: major bleeding and MACE: 1295 vs. 1908 respectively. Most importantly, our meta-analysis shows that there is a treatment interaction in three subgroups: non-smokers, male sex, and treatment with statins.

Two recently published meta-analyses have provided information about the use of aspirin in primary prevention of CVD [39, 40]. Our meta-analysis confirms previous findings and provides additional value with four distinct subgroup analyses and a mortality-adjusted net clinical benefit analysis.

One of the most important findings of our study is the net clinical benefit of aspirin, adjusted for the risk of event-associated mortality, which aims to balance the preventive impact of aspirin on risk for ischemic events such as MI and IS, versus the impact of increased risk of bleeding. The outcome of intracranial hemorrhage is generally worse than the outcome of IS or MI, with the best outcome following a GI bleeding event. Based on previous estimates [31, 32], we weighted hemorrhagic stroke threefold worse than IS. Our weighted analysis provides quantitative assessments of the net clinical benefit of aspirin among primary CVD prevention patients and confirms the result of the crude net clinical benefit estimation. Although models adjusting for event-associated mortality are commonly used [31, 32], weighting one nonfatal event against another is very difficult, as the risks might differ between patients. Therefore, it is still unclear how to properly weight an ischemic event against a bleeding event. Some people with a high risk of having an ischemic event will prefer to take the risk of having a GI-bleed on aspirin, in order to reduce the risk of IS or MI. As there was no significant difference in mortality, intracerebral hemorrhage, or hemorrhagic stroke between aspirin and control, patient preferences should be considered.

Considering upper GI bleeding, which is the most common complication in patients under antiplatelet therapy [41–43], PPIs have been proven effective in the prevention of GI bleeding and are recommended in patients at increased risk for this bleeding [44]. On the other hand, long-term treatment with PPIs is associated with increased risk of community-acquired pneumonia (CAP) [45], bone fractures [45, 46], and enteric infections, mainly by *Salmonella* and *Campylobacter* spp. [45]. Furthermore, PPI-related hypomagnesemia is of clinical significance as it is a known cause of cardiac arrhythmias [45]. Thus, in consideration of the benefits and risks of the respective treatments, the question arises as to whether patients without bleeding risk should receive long-term treatment with PPIs concomitantly with aspirin for primary prevention.

A population of special interest is patients treated with statins. Interestingly, our subgroup analysis comprising 18,000 patients who were concomitantly treated with statins and aspirin showed a benefit in terms of MACE reduction, whereas those treated with aspirin without statins did not. Remarkably, patients treated with aspirin and statins showed the highest RRR of MACE of 12% compared to the overall population and patients with DM. A possible explanation for this interaction might be the consideration that those taking statins are at higher risk for CVD because of hyperlipidemia, and therefore might benefit more. Another possible elaboration might be a direct plaque-stabilizing effect of statins, which, in combination with platelet inhibition by aspirin, improves ischemic outcome. Notably, statins are associated with reduced platelet reactivity and improved response to aspirin [47–52]. However, it is unclear whether the improved response to aspirin under statin treatment is caused directly by statin-platelet interaction, indirectly via reduced levels of lipids [47–49, 51, 52], or by a combination of the two. Elevated cholesterol levels have been linked to decreased aspirin-induced platelet acetylation, explaining the indirect effect of statins on platelet inhibition [53]. Two mechanisms have been identified as being involved in the direct effect of statins on platelets [54]. Administration of atorvastatin resulted in the downregulation of phospholipase A2 (PLA2) (after 24 h) and NOX2 (after 2 h) leading to reduced levels of TxA_2_ and prothrombotic platelet isoprostanes respectively [55]. Based on these findings, early and late antiplatelet effects of statins have been hypothesized [54].

Interestingly, our subgroup analysis showed aspirin use in non-smokers to reduce the risk of MACE by 10%, whereas smokers did not benefit from aspirin treatment. This confirms the result of a previous meta-analysis by Seidu et al., who describe a 30% risk reduction with aspirin in non-smokers [56]. Smoking has been linked to an attenuated antiplatelet effect of aspirin in the past [57–59], and our meta-analysis suggests a possible translation of this phenomenon into clinical practice. In current smokers, a treatment switch from aspirin to the P2Y_12_ receptor inhibitor clopidogrel seems to be an interesting alternative. Smoking is a known inducer of cytochrome P450 (CYP) 1A2, an essential isoenzyme that converts clopidogrel into its active metabolite, and thus may facilitate an adequate platelet inhibition [60]. Studies have demonstrated fewer ischemic events in smokers following clopidogrel administration [61, 62]; however, in primary prevention of CVD, the overall role of clopidogrel has not yet been investigated.

It is crucial to note that our meta-analysis has shown sex differences in aspirin effects. Aspirin showed a reduction of MACE in men but not in women. In contrast, aspirin reduced the risk of stroke in women as shown in a single study, but not in men. Results from previous meta-analyses have also detected a more pronounced effect of aspirin for MACE or MI in men and for stroke in women [5, 63–65]. Although sex differences in aspirin effects are of interest, it is currently unclear how they can be used in clinical decision-making [8, 16, 66].

Another population of special interest is patients with DM. Diabetes increases the risk of CVD, and aspirin is therefore expected to have a greater preventive effect in these patients [67]. In our subgroup analysis comprising over 20,000 patients with diabetes mellitus, aspirin showed a significant 9% RRR in MACE, which confirms the estimate in the overall population. While older guidelines have deemed the use of aspirin reasonable in certain patient populations with diabetes [66], current 2019 guidelines from the ACC/AHA do not specifically comment on the use of aspirin in diabetic patients in primary prevention of CVD [15]. The newly published ESC guidelines on diabetes, pre-diabetes, and CVD have stated aspirin may be used in patients with DM at high/very high risk of CVD and in the absence of clear contraindications (class IIb) [68].

In the general population, the U.S. Preventive Services Task Force’s guideline recommends aspirin for patients based on age and prediction tools such as the 10-year cardiovascular disease calculator [16]. Importantly, these recommendations are given with a moderate evidence level (B and C). The 2019 ACC/AHA guidelines acknowledge the controversy of aspirin in primary prevention of CVD, but state that aspirin might be considered in selected adults aged 40 to 70 who are at higher CV risk but at no increased bleeding risk [15]. Additionally, two cost-utility analyses suggest a clear benefit of aspirin [69, 70]. However, the ESC guidelines on CVD prevention do not recommend the general use of aspirin for the primary prevention of cardiovascular disease [8].

### Limitations

The main limitation is that some studies did not differentiate between ischemic and hemorrhagic stroke. In such cases, the total of “all strokes” was included. The primary endpoint and follow-up periods also differed between some studies; we have adjusted for this in the sensitivity analyses. Another limitation of this meta-analysis was the use of heterogeneous definitions of major bleeding. One study used the GUSTO bleeding classification [17]; most others used a prespecified composite of bleeding events such as GI bleeding and major extracranial bleeding and defined their severity by hospitalization, prolongation of hospitalization, surgery, transfusion requirement, or fatality. The severity and definition of GI bleeding events were often not further detailed.

Furthermore, some trials included in our meta-analysis [25, 27, 28, 30] were performed several decades ago. Since then, there may have been changes in medical standards, the prevalence of risk factors, and access to early diagnostic services.

## Conclusions

The increased risk of major bleeding and lack of reduction of mortality might outweigh the benefits of aspirin in primary prevention of CVD in the overall population. Three patient subgroups: non-smokers, patients treated with statins, and males, had the greatest risk reduction of MACE.

## Additional file

**Additional file 1:** **Figure S1.** Workflow of studies included in the meta-analysis. **Figure S2.** Forest plots depicting the relative risk (RR) of the (A) Primary efficacy outcome (all-cause mortality), (B) MACE and (C) primary safety outcome (major bleeding). **Figure S3.** Forest plots depicting the relative risk (RR) of the (A) Primary efficacy outcome (all-cause mortality), (B) MACE and (C) primary safety outcome (major bleeding). **Figure S4.** Forest plots depicting the relative risk (RR) of (A) extracranial major bleeding, (B) hemorrhagic stroke, (C) gastrointestinal (GI) bleeding and (D) intracranial hemorrhage. **Figure S5.** Forest plot depicting the relative risk (RR) of cancer. **Figure S6.** Forest plot depicting the crude net clinical benefit (NCB) analysis. **Table S1.** Random-effect and fixed-effect models calculated for primary, secondary and exploratory outcomes.

## Data Availability

All data was extracted from already published research and can be accessed by everyone; all publicly available data is cited.

## Abbreviations

ABI: Ankle-brachial index
ARR/I: Absolute risk reduction/increase
CAP: Community-acquired pneumonia
COX-1: Cyclooxygenase-1
CV: Cardiovascular
CVD: Cardiovascular disease
CYP: Cytochrome P450
GI: Gastrointestinal
*I*^2^: Statistical inconsistency test
IS: Ischemic stroke
MACE: Major adverse cardiovascular event
MI: Myocardial infarction
NNT/NNH: Number needed to treat/harm
NO: Nitric oxide
PLA2: Phospholipase A2
PRISMA: Preferred Reporting Items for Systematic Reviews and Meta-Analyses
RCT: Randomized controlled trial
RR: Relative risk
RRR/I: Relative risk reduction/increase
TxA_2_: Thromboxane A_2_
DM: Diabetes mellitus
95% CI: 95% confidence interval
