# Supplementary material for: Aspirin for primary prevention of cardiovascular disease: a meta-analysis with a particular focus on subgroups
Source: BMC Med. 2019 Nov 4;17:198. doi: 10.1186/s12916-019-1428-0 (PMC6827248; doi:10.1186/s12916-019-1428-0)
Supplement: Supplementary file 1 — Additional file 1: Figure S1. Workflow of studies included in the meta-analysis. Figure S2. Forest plots depicting the relative risk (RR) of the (A) Primary efficacy outcome (all-cause mortality), (B) MACE and (C) primary safety outcome (major bleeding). Figure S3. Forest plots depicting the relative risk (RR) of the (A) Primary efficacy outcome (all-cause mortality), (B) MACE and (C) primary safety outcome (major bleeding). Figure S4. Forest plots depicting the relative risk (RR) of (A) extracranial major bleeding, (B) hemorrhagic stroke, (C) gastrointestinal (GI) bleeding and (D) intracranial hemorrhage. Figure S5. Forest plot depicting the relative risk (RR) of cancer. Figure S6. Forest plot depicting the crude net clinical benefit (NCB) analysis. Table S1. Random-effect and fixed-effect models calculated for primary, secondary and exploratory outcomes. [file 12916_2019_1428_MOESM1_ESM.docx]

**Additional files / Supplementary appendix:**

**Figure S1.** Workflow of studies included in the meta-analysis.

**Figure S2.** Forest plots depicting the relative risk (RR) of the (A) **Primary efficacy outcome (all-cause mortality),** (B) **MACE and** (C) **primary safety outcome (major bleeding)**.

**Figure S3.** Forest plots depicting the relative risk (RR) of the (A) **cardiovascular mortality,** (B) **myocardial infarction and** (C) **ischemic stroke**.

**Figure S4.** Forest plots depicting the relative risk (RR) of (A) **extracranial major bleeding**, (B) **hemorrhagic stroke**, (C) **gastrointestinal (GI) bleeding** and (D) **intracranial hemorrhage**.

**Figure S5.** Forest plot depicting the relative risk (RR) of **cancer**.

**Figure S6.** Forest plot depicting the crude net clinical benefit (NCB) analysis.

**Table S1.** Random-effect and fixed-effect models calculated for primary, secondary and exploratory outcomes.

| Event | random-effect model | fixed-effect model |
| --- | --- | --- |
| Myocardial infarction | RR 0.86; 95% CI, 0.77-0.95; p=0.005; I^2^=50% | RR 0.86; 95% CI, 0.80-0.92; p<0.0001; I^2^=50% |
| Ischemic stroke | RR 0.90; 95% CI, 0.82-0.99; p=0.03; I^2^=17% | RR 0.90; 95% CI, 0.83-0.98; p=0.01; I^2^=17% |
| MACE | RR 0.91; 95% CI, 0.86-0.95; p<0.0001; I^2^=0% | RR 0.91; 95% CI, 0.86-0.95; p<0.0001; I^2^=0% |
| Cancer | RR 1.05; 95% CI, 0.87-1.26; p=0.64; I^2^=54% | RR 1.02; 95% CI, 0.94-1.11; p=0.61; I^2^=54% |
| Cardiovascular death | RR 0.99; 95% CI, 0.90-1.08; p=0.75; I^2^=0% | RR 0.99; 95% CI, 0.90-1.08; p=0.75; I^2^=0% |
| All-cause death | RR 0.98; 95% CI, 0.93-1.02; p=0.26; I^2^=0% | RR 0.98; 95% CI, 0.93-1.02; p=0.26; I^2^=0% |
| Major bleeding | RR 1.46; 95% CI, 1.30-1.64; p<0.00001; I^2^=31% | RR 1.42; 95% CI, 1.31-1.54; p<0.00001; I^2^=31% |
